# Prevention of arterial catheter-related bloodstream infections: current evidence and future directions

**DOI:** 10.1186/s13054-026-06153-z

**Published:** 2026-07-06

**Authors:** Viviane Donner, Christine D. Sadeghi, Gaud Catho, Jean-François Timsit, Giovanni Satta, Claire M. Rickard, Leonard Mermel, Niccolò Buetti

**Affiliations:** 1 Department of Emergency Medicine, Fribourg Cantonal Hospital, Ch. des Pensionnats 2-6, Fribourg, CH 1700 Switzerland; 2 Department of Intensive Care Medicine, Valais Hospital, Av. Grand-Champsec 80, Sion, CH 1951 Switzerland; 3 Division of Intensive Care, Department of Anaesthesiology, Clinical Pharmacology, Intensive Care and Emergency Medicine, Geneva University Hospital, Rue Gabrielle-Perret- Gentil 4, Geneva, CH 1211 Switzerland; 4 Department of Intensive Care Medicine, Fribourg Cantonal Hospital, Ch. des Pensionnats 2-6, Fribourg, CH 1700 Switzerland; 5 Critical Care Complex, Counties Manukau, Middlemore Hospital, 100 Hospital Road, Ōtāhuhu, 1640 Auckland New Zealand; 6 Infection Control Program, Faculty of Medicine, Geneva University Hospitals, World Health Organization Collaborating Centre, Geneva, Switzerland; 7 Division of Infectious Diseases, Central Institute, Valais Hospital, Sion, Switzerland; 8 APHP Bichat University Hospital Medical and Infectious Diseases ICU, Paris, 75018 France; 9 Centre for Clinical Microbiology, Division of Infection and Immunity, University College London, London, UK; 10 Herston Infectious Diseases Institute, Metro North Health, RBWH Herston, Brisbane, 4006 Australia; 11 School of Nursing, Midwifery and Social Work, Frazer Institute, The University of Queensland, RBWH Herston, Brisbane, 4006 Australia; 12 Alliance for Vascular Access Teaching and Research, Schools of Nursing and Midwifery and Pharmacy and Medical Science, Griffith University, Southport, Gold Coast, 4222 Australia; 13 Department of Epidemiology & Infection Prevention, Brown University Health, Providence, Rhode Island USA; 14 Division of Infectious Diseases, Rhode Island Hospital, Providence, Rhode Island USA; 15 INSERM, IAME UMR 1137, Université de Paris, Paris, 75018 France

**Keywords:** Arterial catheter, Peripheral arterial catheter, Catheter related bloodstream infection, Catheter related infection prevention

## Abstract

Arterial catheters (ACs) are essential devices widely used for hemodynamic monitoring and blood sampling. Whilst their infectious risk is well-known, the surveillance and identification of arterial catheter-related bloodstream infections (AC-CRBSI) remains underappreciated, and guidelines recommending best practices to prevent infection are few and inconsistent. Most evidence for AC infection prevention is inferred from studies focused on central venous catheter infection prevention. Few studies have directly explored optimal infection prevention strategies for ACs. For AC-CRBSI prevention, several strategies are valuable during insertion and maintenance of the catheter: minimisation of catheter-days by appropriate catheter stewardship, procedural training of providers, standardisation of insertion packs to promote adherence to an insertion bundle, cutaneous antisepsis with an alcohol-based 2% chlorhexidine solution during insertion and maintenance, chlorhexidine impregnated dressings, change of dressing every 7 days unless visibly disrupted or soiled, hand hygiene, disinfection of the access port and change of administration sets every 7 days. Other interventions to prevent AC-CRBSI are more controversial and necessitate further research such as the choice of the insertion site (femoral vs. radial), the type of sterile barrier precautions and their cost effectiveness, the infectious risk related to ultrasound guidance, the optimal catheter securement device, the utility of needleless connectors and closed loop blood sampling systems and the effectiveness of scheduled catheter changes. Arterial catheters demonstrate an ongoing risk of contamination throughout their lifespan, from insertion to removal. A comprehensive prevention strategy should address the entire arterial catheter lifecycle and is most effective when implemented as a bundled approach. This review summarises current knowledge on AC-CRBSI prevention during the entire lifecycle of the catheter and identifies priorities for future research.

**Graphical abstract:**

**Figure:**
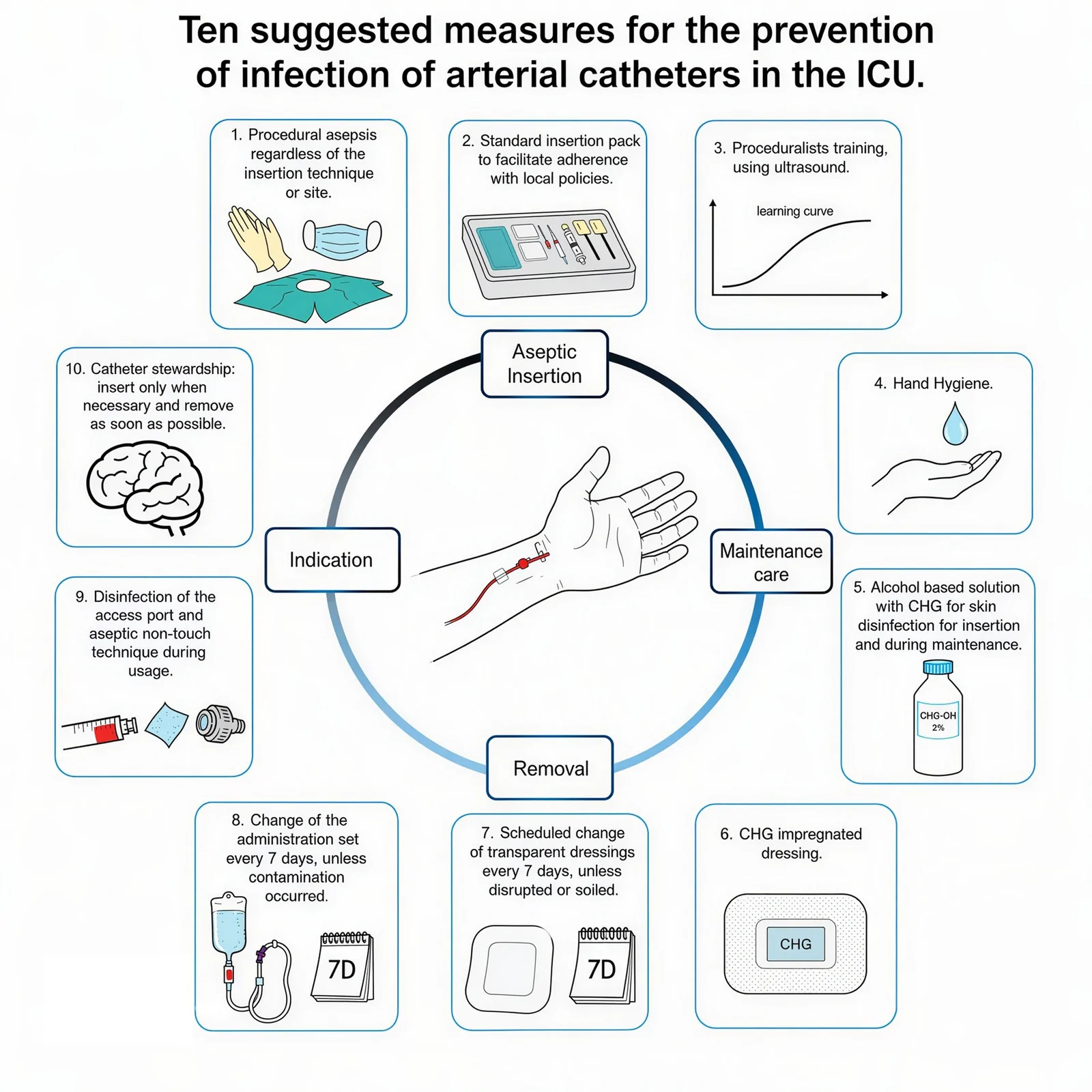

## Background

Arterial catheters (AC) are inserted to facilitate hemodynamic monitoring and frequent blood sampling in operative and critical care settings. Each year, millions of ACs are inserted worldwide, and their use may lead to complications such as arterial occlusion, bleeding, thrombosis, dislodgement and infection [1]. In particular, ACs pose a risk of bloodstream infection, and due to their ubiquity in the intensive care unit (ICU), they have the potential to significantly impact healthcare costs and patient outcomes.

The risk of AC related bloodstream infections (AC-CRBSI) has been known for decades, and approaches that of central venous catheters (CVC) [2–6]. However, this risk remains underappreciated [7] particularly among critical care staff [6, 8, 9], and largely disregarded in common surveillance systems compared to central line infections [10, 11]. Reasons why ACs have historically been overlooked include the perception that ACs are short-term devices that pose little threat, and the focus on CVCs as the potential source of infection in patients with both devices.

Each stage of the AC’s lifecycle, from indication to removal, during insertion and maintenance, presents an opportunity for preventive measures to reduce the risk of AC-CRBSI. Implementation of care bundles have been shown effective in decreasing the incidence of central line-associated bloodstream infections (CLABSI) [12]. However, research focusing on AC care bundles is scarce, thus good practice guidelines on the management of ACs are nonspecific and rely substantially on evidence inferred from CLABSI prevention [13]. ACs are distinct intravascular devices that may require specific infection prevention measures.

This narrative review aims to highlight opportunities for prevention of AC-CRBSI throughout the catheter lifecycle. After summarising current evidence specific to ACs, we suggest a set of effective strategies and explore future research directions to narrow the knowledge gap in AC infection prevention.

## Main text

### Pathophysiology of arterial catheter infection

AC-CRBSI arises from the interaction between bacterial colonisation of the catheter and host factors that ultimately lead to bloodstream infection. AC-CRBSI is mainly acquired by two routes; the extraluminal route via migration of skin flora from the insertion site along the external surface of the catheter, and the intraluminal route via contamination of the catheter hub during manipulation [14]. Catheter colonisation is a prerequisite for the development of most CRBSIs, and has long been used as a surrogate marker for infection in research regarding catheter infections. However, recent literature questions colonisation as a valid surrogate to establish the effectiveness of infection prevention interventions [15]. Some historical data indicates that, in the absence of an aseptic insertion bundle, the extraluminal route accounts for most AC-CRBSI acquisitions [5].

### Definition and diagnosis of arterial catheter related bloodstream infection

AC-CRBSI is a bloodstream infection directly attributable to an AC. To confirm AC-CRBSI, evidence is required that the microbiology of the bloodstream infection (BSI) matches that of the AC, as demonstrated by catheter-tip culture or by differential time to positivity (dTTP) [16]. This definition establishes a causal relationship between the AC and the BSI and is used for clinical and research purposes but only infrequently as a surveillance metric.

Some clinicians remain reluctant to draw blood cultures from the AC due to concerns about contamination, even though blood culture collection from ACs has been shown to be non-inferior to venipuncture in critically ill patients [17, 18]. This is in contrast with blood cultures drawn from indwelling venous catheters, which show higher contamination rates as compared to venipuncture [19, 20]. Given the difficulty of peripheral venipuncture in critically ill patients, blood cultures obtained from all indewelling intravascular catheters remain clinically relevant to avoid delay in initiation of antibiotic therapy in septic patients. Careful, context-informed interpretation is essential.

Among clinicians, the perceived risk of infection may likely influence the threshold for culturing the AC and may lead to underreporting and/or an unappreciation of ACs as the source of infection. In critical care patients with an unknown source of infection, at least one set of blood cultures should be obtained from the AC in addition to those drawn percutaneously and from the CVC. AC tip culture can help confirm the source of infection when the catheter is removed for suspected infection [16]; however, it has limitations and potential pitfalls that must be considered during interpretation [21, 22].

### Surveillance of AC-CRBSI

AC infections are overlooked by popular surveillance metrics used for quality monitoring in ICUs such as CLABSI [23]. Because many patients are concomitantly equipped with both an AC and a CVC, surveillance using the CLABSI metric may falsely attribute a bloodstream infection to the CVC when, in fact, the AC is the primary source.

Catheter-associated bloodstream infection (CABSI) is a broader metric that, in addition to CVCs, captures BSI associated with any other intravascular catheter such as peripheral intravenous catheters (PIVC), peripherally inserted central catheters (PICC) and ACs. Causal attribution to a particular intravascular device by catheter tip culture or dTTP is not always necessary for a diagnosis of CABSI. This metric has previously been used in surveillance programs of intravascular devices including ACs [1, 24].

Hospital onset bacteraemia (HOB) is emerging as a novel automatable surveillance metric that refers to a positive blood culture, with a recognised pathogen, occurring two to four days or more after hospital admission, depending on the definition [25, 26]. HOB includes all nosocomial bacteraemia and fungaemia cases regardless of the source. Previous data shows that AC use is associated with an increased risk of HOB [27], which highlights the potential of this new metric for surveillance of AC infections and the monitoring of the effect of new preventive strategies. Key limitations of this metric are the absence of a universal definition, lack of source attribution and limited sensitivity to AC-specific prevention practices.

### Epidemiology of AC-CRBSI

Interpretation of epidemiological factors affecting AC infections are challenging because endpoints used in past studies to define catheter infection have been heterogeneous. Reporting and surveillance remain poorly standardised [28].

In past studies, the incidence density of AC-CRBSI has been shown to be similar to the incidence density of CVC-CRBSI [5, 6, 29, 30]. Many studies that provide epidemiological data on AC-CRBSI were conducted prior to routine implementation of effective catheter care bundles in the ICU. Data from more recent studies report an incidence of AC-CRBSI ranging from 0.1 to 0.2/1000 catheter days [31, 32]. SPIADI is a surveillance programme encompassing a majority of French ICU beds, and have reported that ACs account for 30% of documented CRBSIs [ 33]. The reported incidence ranged from 0.3 to 0.4/1000 ICU-days from 2020 to 2024, only slightly lower than the incidence of CVC-CRBSI for the same period.

### Key features of arterial catheters

ACs exhibit several features that distinguish them from CVCs during the insertion and maintenance phases.

**Fig. 1:**
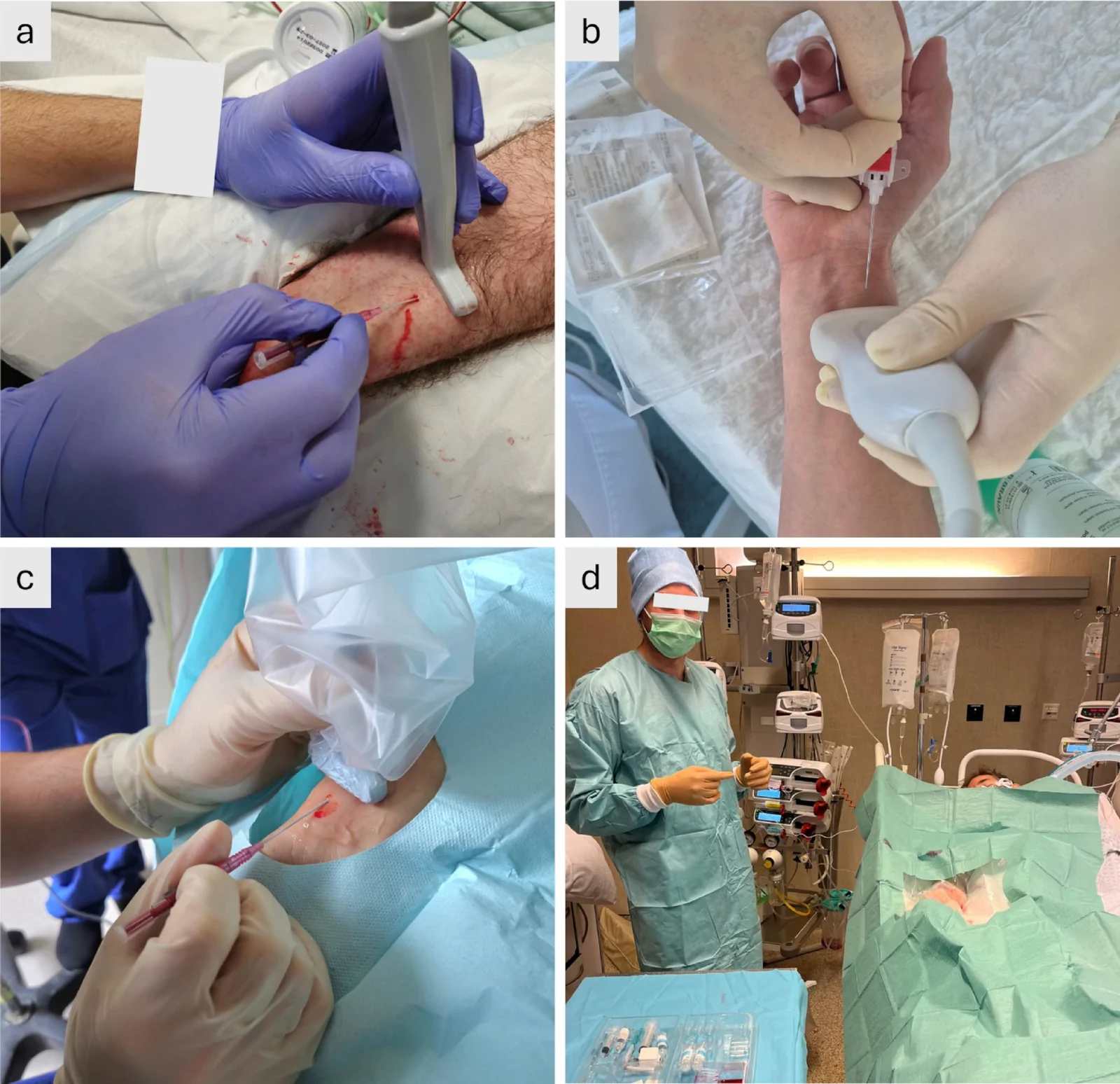
Spectrum of infection prevention practices in critical care units for insertion of radial catheters (**a**) catheterisation using an over-the-needle technique with ultrasound guidance using non sterile gloves (no insertion bundle) (**b**) catheterisation using an over-the-needle technique with ultrasound guidance using sterile gloves and an aseptic non-touch technique (ANTT) (**c**) catheterisation using an over-the-needle technique with ultrasound guidance using sterile barrier precautions (SBP) (**d**) catheterisation with the Seldinger technique without ultrasound guidance, using maximal sterile barrier precautions (MSBP)

Technique of insertion depends on the insertion site. Femoral AC insertion necessitates the Seldinger technique, which uses a free guidewire inserted into the needle after puncturing the vessel, akin to a CVC insertion. Radial AC insertion can be performed using several alternative approaches such as the direct puncture or over-the-needle technique which closely resembles peripheral iv cannulation (Fig. 1).

In the postinsertion phase, ACs are used for blood sampling requiring staff to frequently access the catheter hub. This may explain why in comparison to CVCs, ACs exhibit a higher risk of colonisation after 7 days, probably due to the frequent access of the ACs with breaches in aseptic technique leading to endoluminal colonisation [3, 30].

Regarding dwell time, many ACs are placed in the operating theatre for short-term monitoring and typically removed after a brief postoperative course in the ICU. In this non critically ill population with short catheter dwell time, the infectious risk is low and may have historically led to underestimating ACs as a potential major source of bloodstream infection in ICU patients [4, 34, 35]. However, the dwell time of ACs inserted in the ICU is longer than those placed in operating theatre with a median dwell time of 6 to 7 days [7, 30, 36], approaching the dwell time of a CVC in the ICU setting [3, 31, 37].

Finally, ACs exhibit a high all cause failure rate (including suspected infection, blockage, dislodgement and lack of haemodynamic trace) affecting roughly one quarter of ACs [1, 38]. In the ADVANCED trial, although infection rates were identical, dysfunction of ACs was much more frequent than for CVCs and approached dysfunction rates of PIVCs [32]. AC failure occurs most often during the first 7 days and frequently leads to AC reinsertion [1]. Factors associated with AC failures are not yet fully elucidated but may include insertion technique, catheter position (distal vs. proximal), securement, catheter size, device type and insertion site [1, 39].

### Past and current recommendations

In 2002, the U.S. Centers for Disease Control and Prevention (CDC) guidelines for the prevention of catheter-related infection stated that ACs are rarely a source of CRBSI [40]. With accumulating evidence that ACs are a potential major source of sepsis, CDC guidelines were updated in 2011 and provided more explicit guidance on AC infection prevention practice [41].

Current guidelines on infection prevention of ACs remain heterogenous especially regarding insertion bundles, while recommendations for postinsertion catheter care are more consistent. Variation in insertion bundles may be explained because ACs lie conceptually somewhere between PIVCs and CVCs, particularly in radial insertions. Depending on local practice, insertion bundles can therefore vary between a CLABSI prevention bundle using maximal sterile barrier precautions (MSBP) and a procedure resembling a PIVC insertion.

Tables 1 and 2 summarise and compare recommendations for the prevention of AC-CRBSI from key guidelines [41–45].

**Table 1:** Comparison of recommendations on infection prevention at insertion of arterial catheters

	CDC/IDSA 2011 (update 2017)	NICE 2012	EPIC3 GUIDELINES 2014	SRLF 2020	WHO 2024
**Device stewardship**	Remove catheter as soon as no longer necessary	/	Remove catheter as soon as no longer necessary or when complications occur. Reminders to review the continuing use or prompt the removal of intravascular devices	Remove catheter as soon as no longer necessary	/
**Site selection**	Peripheral sites^a^ are preferred over central sites^b^	/	/	/	/
**Barrier precautions**	SBP + cap for peripheral sites^a^ MSBP for central sites^b^	/	/	MSBP regardless of site or insertion technique	SBP + sterile gown regardless of site or insertion technique
**US guidance**	/	/	/	Femoral and radial catheters should be inserted under US guidance to decrease mechanical complications	/
**Training**	Formal training and continuous education on catheter insertion for clinicians	/	Formal training and continuous education on catheter insertion for clinicians. Quality improvement programmes recommended	Quality improvement programmes recommended	Formal training and continuous education on catheter insertion for clinicians
**Cutaneous antisepsis**	> 0.5% CHG	CHG – 70% alcohol	2% CHG – 70% alcohol	2% CHG – alcohol	Skin antisepsis recommended (either a CHG-containing or alternative antiseptic)
**Standardised insertion pack**	/	/	/	Recommended	Recommended

*CDC/IDSA* Centers for Disease Control and Prevention/Infectious Diseases Society of America; *NICE* National Institute for Health and Care Excellence; *SRLF* Société de Réanimation de Langue Française; *WHO World Health Organization; MSBP* Maximal sterile barrier precautions (cap, mask, sterile gown, sterile gloves, full-body sterile drape); *SBP* Sterile barrier precautions (mask, sterile gloves and fenestrated drape); *US* Ultrasound; *CHG* Chlorhexidine gluconate. ^a^ Peripheral sites: radial, brachial, dorsalis pedis ^b^ Central sites: femoral and axillary sites

**Table 2:** Comparison of recommendations on infection prevention during maintenance and removal of arterial catheters

	CDC/IDSA 2011 (update 2017)	NICE 2012	EPIC3 GUIDELINES 2014	SRLF 2020	WHO 2024
**Hand hygiene**	Hand hygiene before and after access, repair, dressing change or removal	Hand hygiene before and after access or dressing change	Hand decontamination and aseptic technique during catheter manipulation, catheter site dressings and system access	Hand hygiene at insertion and for all catheter manipulations	Hand hygiene during catheter maintenance, access and removal practices
**Securement**	Use a sutureless securement device to reduce the risk of infection	**/**	**/**	**/**	**/**
**Dressing type**	/	CHG-impregnated transparent semipermeable dressing.* Sterile gauze dressing only if perspiring profusely or if insertion site is bleeding/oozing	Transparent semipermeable dressing Sterile gauze dressing only if perspiring profusely or if insertion site is bleeding/oozing	CHG-impregnated transparent semipermeable dressing	A formal sterile dressing protocol should be used (not specified)
**Dressing changes**	/	Change gauze dressing every 24 h, or sooner if dressing soiled or no longer intact Replace with transparent semi-permeable dressing as soon as possible Change the transparent dressing every 7 days, or sooner if soiled or no longer intact	Change gauze dressing when inspection of the insertion site is necessary or if dressing soiled or no longer intact Replace with a transparent semi-permeable dressing as soon as possible Change the transparent dressing every 7 days, or sooner if soiled or no longer intact	Change the transparent dressing every 7 days, or sooner if soiled or no longer intact	/
**Usage/access**	Minimise the number of manipulations. Prefer a closed system (continuous flush) rather than an open one (stopcocks/syringe) If using a diaphragm rather than a stopcock to access system, decontaminate access point before use	Decontaminate access point with CHG – 70% alcohol before and after use Consider CHG-aqueous solution if alcohol prohibited with catheter	Decontaminate access point with 2% CHG – 70% alcohol before use	Decontaminate access point before use If open system, manipulate in a sterile compress or alcohol compress	A formal sterile or aseptic protocol should be used to access ACs (not specified)
**Administration sets**	Continuous administration sets and all other components of the system need not be changed more frequently than **96-hour** intervals	Continuous administration sets need not be changed more frequently than **72-hour** intervals	Continuous administration sets need not be changed more frequently than **96-hour** intervals. Use normal saline, no benefit for heparin solutions	Continuous administration sets need not be changed more frequently than **96-hour** intervals	/
**Scheduled catheter changes**	Do not routinely replace ACs, only if clinically indicated	/	ACs should be re-sited when clinically indicated and not routinely	/	/

*CDC/IDSA* Centers for Disease Control and Prevention/Infectious Diseases Society of America; *NICE* National Institute for Health and Care Excellence; *SRLF* Société de Réanimation de Langue Française; *WHO World Health Organization; CHG* chlorhexidine gluconate; *AC* arterial catheter; *NICE guideline update on CHG impregnated dressings [46]

### Current practice regarding bundle of care for arterial catheters

After the publication of the CDC infection prevention guidelines in 2011, surveys highlighted a variable adherence to prevention measures for peripheral ACs [8, 47]. An Australian audit revealed that sterile gloves are used by only 57% of practitioners when inserting ACs in a paediatric ICU [38]. A multicentre survey in China revealed significant heterogeneity across hospitals regarding antiseptic technique and perception of infection prevention during catheter insertion, maintenance and removal [9]. A recent ANZICS observational study also showed significant variation in the management of catheter care after insertion (dressing types and dressing care as well as AC scheduled removal) [48]. Apart from these few surveys, global data on current practice in ICUs is unavailable.

### Evidence on the prevention of infection for arterial catheters

#### Catheter stewardship

By limiting catheter days, judicious AC insertion and early removal are key for the prevention of AC-related complications, including infectious risk [49, 50]. Overuse of ACs is a concern.

As with other monitoring devices in the ICU, AC insertion is not associated with improved patient outcomes [51–53]. Rather, ACs correlate with increased severity of illness, reflecting a need for frequent monitoring in more critical patients [48]. Recently, a large RCT demonstrated that a deferred strategy (i.e. avoiding AC insertion unless the patient met certain prespecified safety criteria) was safe and non-inferior to an early AC insertion strategy with regards to 28-day mortality in patients with shock [54].

Recent data suggests that quality initiatives using cognitive aids or medical record nudging to encourage routine device review are potentially effective in preventing medical device-related infections [55, 56]. These approaches could be adapted for the prevention of arterial catheter–related bloodstream infections by targeting a reduction in catheter-days.

*ACs should be inserted only when clinically indicated and removed as soon as no longer necessary*.

#### Site selection

Radial access is the routine site for peripheral AC placement in the critical care and theatre setting due to ease of access. The femoral site may account for up to 36% of ACs [37] and is preferred in case of shock requiring emergency access, for hemodynamic monitoring (central blood pressure, transpulmonary thermodilution), or in case of radial arterial access failure. Outside of radial and femoral sites, alternative sites (e.g., dorsalis pedis, brachialis, axillary) are infrequently used (< 10%) [1], except in very young children.

Femoral and radial catheters differ in aspects such as insertion technique, natural history of the catheter, maintenance practice and host factors that may account for challenging confounding factors when assessing site specific infectious risk retrospectively [4, 57].

Accounting for some known confounding variables, post-hoc analyses using data from three large randomized controlled trials (RCTs) on cutaneous antisepsis and dressings [58–60] revealed that CRBSI risk is similar for catheters inserted in the femoral compared to the radial artery although femoral catheters had a higher risk of colonisation [61] and a higher proportion of gram negative bacilli CRBSI [62]. The higher risk of catheter tip colonisation for the femoral site has been observed in multiple studies [2, 3, 30] but recent data questions colonisation as an appropriate surrogate for CRBSI [15]. No randomised trials have been undertaken of site placement in ACs with infection endpoints. Recently released trials comparing sites for arterial catheterisation have not assessed this endpoint [63, 64].

Apart from the insertion site, the location of the catheter may also influence infectious risk. Recent data suggest that radial ACs inserted distally near the wrist may exhibit a higher risk of AC-CRBSI than ACs placed farther away from the joint [65].

*There is insufficient data to recommend any given insertion site over another as an effective preventive measure for AC-CRBSI*.

#### Barrier precautions

Barrier precautions are a controversial aspect in AC infection prevention. In practice, barrier precautions at insertion of ACs are inconsistently applied and commonly considered of variable utility depending on the cannulation technique and the site of insertion. Figure 1 illustrates the wide spectrum of practice at insertion, from total lack of an insertion bundle to MSBP.

Heterogeneity in guidelines may account for the wide variation in the application of insertion bundles. CDC guidelines recommend site-specific barrier precautions, with MSBP for femoral and axillary site catheterisation, as these are considered central sites [41]. In contrast, the French Society of Intensive Care Medicine (SRLF) recommend applying MSBP regardless of the site or insertion technique [44]. The WHO recently released a CRBSI prevention guideline including recommendations for ACs [45]. WHO recommends using a “sterile technique”, which differs from MSBP in that it does not require a cap, and that the drape covers only the area surrounding the insertion site rather than the entire body. Some recent guidelines still omit guidance for ACs [66]. In addition, current guidelines rarely account for ultrasound (US) which is increasingly used for AC insertion and requires higher attention to infection prevention including the use of sterile probe covers and device disinfection.

There are very few direct data on the effectiveness of barrier precautions for the prevention of AC infection. More than two decades ago, a monocentric RCT was carried out on the use of barrier precautions at AC insertion and failed to show superiority of MSBP over aseptic non-touch technique (ANTT) with sterile gloves [67]. In addition, only radial catheterisations using an over-the-needle technique were randomized, excluding other insertion sites and those using the Seldinger technique. This underpowered trial is the only one to date that assessed the impact of barrier precautions for AC insertion on risk of infection.

In past studies, MSBP have been shown to be both effective and cost-effective for the prevention of CLABSI when implemented as part of a care bundle for CVCs [68, 69]. The relative value of individual components of the insertion bundle remains however largely unknown. No similar study to date has investigated these measures for AC insertion specifically.

*Procedural asepsis as part of a care bundle during insertion is effective in limiting extraluminal colonisation and thus may be effective for the prevention of AC-CRBSI. Optimal barrier precautions across various insertion techniques and settings of ACs remain uncertain*.

#### Ultrasound guidance for arterial catheterisation

There is now high-level evidence to support real-time ultrasound guidance for radial and femoral arterial cannulation in both adult and paediatric populations over a palpation technique. Ultrasound guidance is endorsed as a GRADE 1 recommendation by international guidelines on vascular access regardless of the site of insertion [70, 71].

In current critical care practice, ultrasound guidance for AC insertion is used marginally in adults (9–24% of all AC insertions) [1, 72, 73] and is more common in paediatric ICU patients [38]. A 2023 French study found that only 16% of clinicians use ultrasound guidance systematically for AC insertion and 21% use it rarely [73].

Ultrasound guidance increases first attempt success, reduces the number of mechanical complications and the procedural time [74–76] which may all reduce infection risk. On the other hand, ultrasound guidance introduces additional manipulations that may increase the risk of contamination at insertion, thus potentially increasing the risk of catheter related infection. Prospective studies assessing the effect of ultrasound guidance for arterial catheterisation have largely disregarded infectious risk. A post hoc analysis of 2 RCTs did not show an effect of ultrasound guidance at insertion on frequency of AC colonisation and AC-CRBSI [72].

*Ultrasound guidance should be used for arterial cannulation independent of site selection to reduce mechanical complications and procedural time. Its effect on infectious risk remains unknown*.

#### Training of providers

A significant proportion of providers of US-guided vascular access fail to comply with infection prevention policies [73] highlighting that sterile procedural technique is challenging to maintain when using ultrasound for vascular access; this is likely remediable with high volume inserters and local processes to ensure competence.

Several studies have demonstrated that formal training in infection prevention bundles – both for vascular access and catheter maintenance – are effective [77]. Simulation based education programmes for CVC insertion under real-time ultrasound guidance with MSBP have been shown to be both cost-effective and effective in reducing CLABSI [78–80]. Formal vascular access training, ideally including a simulation-based component is important for novice providers [81].

*Clinicians’ training focusing on sterile procedural technique with ultrasound and continuous quality improvement programs are valuable strategies for infection prevention*.

#### Cutaneous antisepsis

Cutaneous antisepsis is an effective strategy to reduce CRBSI. Common cutaneous antiseptic products include chlorhexidine gluconate-based solutions (CHG), povidone-iodine (PVI) and alcohol-based solutions. Alcohol and PVI provide immediate and short-term antimicrobial activity, while CHG offers prolonged antimicrobial activity.

A Cochrane review summarised available evidence on cutaneous antisepsis for the prevention of CRBSI in intravascular devices [82]. In this systematic review, ACs were underrepresented with only three RCTs including these devices [83–85]. Due to the timing of publication, the CLEAN trial [60] was not included in the Cochrane review. This multicentric study is currently the largest study on antiseptic regimens for intravascular devices of which approximately half were ACs. It compared skin antisepsis with a CHG-alcohol solution (2% CHG–70% isopropyl alcohol) and a PVI-alcohol solution (5% PVI–69% ethanol) to prevent CRBSI in the ICU, both at insertion and for each dressing change. The CHG-alcohol solution showed significant superiority over PVI-alcohol in infection prevention. The risk of CRBSI was also significantly reduced in the subgroup of ACs. The benefit for 2% CHG solution was observed at the cost of increased skin sensitivity reactions.

CHG bathing for ICU patients is a preventive strategy that has been shown effective to reduce CLABSI and ICU acquired bacteriaemia but its effect on AC-CRBSI remains unknown [86].

*Cutaneous antisepsis with a 2% CHG-alcohol solution is effective for infection prevention during insertion and maintenance of ACs*.

#### Standardised insertion pack

Some studies have examined bundles of care that include the use of a standard procedure pack or cart, but solely for the prevention of CLABSI [69, 80, 87]. These grouped interventions have been shown to be effective in decreasing the incidence of CLABSI. One single study examined the use of standardised packs for PIVCs, and found that use of a standardised kit was associated with a lower rate of phlebitis, longer lifespan of the PIVC, and decreased costs [88]. Direct data regarding ACs are lacking.

Standardised packs not only facilitate training and education but also simplify adherence to guidelines, minimize contamination risks by providing preassembled items, and enhance procedural efficiency. The WHO as well as the SRLF have recommended the use of standard insertion packs as a good practice statement [44, 45]. To date, the ideal pack contents have not been defined. Future research could address this topic.

*By analogy with CVCs and PIVCs, the use of standardised packs for AC insertion is a valuable strategy to facilitate adherence to guidelines and local protocols while improving procedural efficiency*.

#### Securement

Securement devices provide protection against accidental removal, dislodgement, and micro-movements of the catheter which may help to prevent catheter failure and infection. Different securement devices are available and are generally either sutures or sutureless securement devices (SSD). Out of international guidelines, only the CDC has previously recommended to prefer SSD for securement of intravascular catheters to reduce the risk of infection [41]. This recommendation is largely based on a monocentric study on peripherally inserted central catheters (PICC) [89]. A recent Cochrane review examined the question of securement devices and dressings in preventing all-cause complications of ACs (AC failure, AC-CRBSI, and other adverse events), and was unable to find robust evidence supporting any particular kind of securement device over another [90].

*While effective securement is important, no particular securement device can be recommended over another for the prevention of AC-CRBSI*.

#### Dressing type

Several different types of dressings are available for ACs, including non-occlusive gauze dressings and occlusive transparent dressings (simple or impregnated).

CHG-impregnated dressings, either with the use of an impregnated sponge or an integrated gel pad, have been shown effective to reduce CRBSI in short term catheters in a meta-analysis including 9 RCTs [91]. More than half of the patient population included in this meta-analysis is provided by two landmark trials evaluating optimal dressing protocols for the prevention of catheter-related infections in critically ill patients [58, 59]. DRESSING1 included CVCs and ACs and showed that CHG-impregnated sponge dressings are superior to standard non-antimicrobial dressings for the prevention of catheter-related infection in a setting of a low basal infection rate [58]. In a sensitivity analysis this finding was inconclusive for ACs [58]. DRESSING2 compared three types of transparent dressings (CHG-gel dressing, highly adhesive non-antimicrobial dressing and standard breathable dressing). It showed that CHG-gel dressings placed at catheter insertion significantly reduce the risk of CRBSI compared to non-CHG dressings. In the subgroup analysis of ACs, the risk reduction was significant for colonisation but not for CRBSI [59].

A secondary analysis of these trials compared both types of CHG impregnated dressings (sponge vs. gel dressings) and showed similar infectious risk [92]. CHG-gel dressings may have a higher risk of contact dermatitis [58, 59] as compared to sponge dressings but allow for inspection of the insertion site [92]. CHG-gel dressings have been widely adopted in ICU due to their ease of use.

Gauze dressings and simple transparent occlusive dressings have shown equivalent catheter infection rates [93]. Impregnated dressings are therefore considered superior to gauze dressings for AC-CRBSI prevention, although direct comparison is lacking. Gauze dressings are a possible option, especially in case of profuse perspiration or oozing of the insertion site. However, they have the disadvantage of being non-occlusive and opaque, therefore not allowing for regular visual inspection of the insertion site and requiring more frequent changes.

CHG impregnated dressings have been shown to further reduce AC infection when added to an existing preventive bundle [24] and are recommended to be routinely used for short-term intravascular catheters [41, 46].

*CHG-impregnated dressings are effective as part of a bundle to prevent AC-CRBSI*.

#### Dressing changes

The risk of dressing disruptions increase with duration of catheter maintenance time and may be an important risk factor for catheter-related infections through the extraluminal route [57]. Therefore, it is usually recommended to include dressing integrity in catheter care bundles [41–44]. Highly adhesive dressings have been shown to reduce the rate of disruption without conferring additional benefits for infection prevention [59]. Scheduled changes of unsoiled occlusive transparent AC dressings every 7 days has been shown to be non-inferior to a change every 3 days as well as reduce the total number of dressing changes [58]. Changing gauze dressings every 1–2 days is recommended to allow regular inspection of the insertion site and appropriate catheter care [42, 43, 45].

*Unless soiled or disrupted, scheduled change of occlusive transparent AC dressings every 7 days is safe*.

#### Usage and access

After insertion, intraluminal contamination of the AC from breaches in aseptic technique when accessing the hub is an important source of infection [94]. Although increased frequency of accessing ACs is thought to be a significant contributor to AC-CRBSI, duration since insertion seems to be the main factor associated with risk of colonisation and AC-CRBSI [95, 96].

Important preventive measures to reduce hub contamination are hand hygiene before any manipulation of the AC, as well as disinfection of the access hub before use and an aseptic non-touch technique during access [97]. Hand hygiene promotion and hub disinfection are proven strategies to reduce the rate of CLABSI as part of a bundle [69, 97, 98], but no evidence specific to AC-CRBSI is available.

The design of the AC access system, such as needleless connectors, may also influence the rate of intraluminal hub contamination, but most studies performed to assess different systems were underpowered to convincingly demonstrate the superiority of any design for the prevention of AC-CRBSI [99–102]. A meta-analysis has shown that closed-loop transducer systems may be advantageous over open systems to reduce iatrogenic blood loss and AC colonisation [103].

*Thorough hand hygiene, disinfection of the access port and aseptic non-touch technique during usage are key to reducing AC-CRBSI*.

#### Administration sets

Administration sets are composed of a bag of pressurised fluid, a pressure transducer for hemodynamic monitoring and a connecting system with the catheter hub to allow for blood sampling.

The fluid content of the set is commonly normal saline (NaCl 0.9%). Different options have been studied, including various heparin solutions. Some studies and one meta-analysis show a possible benefit of heparin solutions to prolong the lifetime of an AC [104], but none have examined the effect of different solutions on infectious risk.

Scheduled replacement of the administration set could lower the infectious risk by decreasing exposure to contaminated sets and flush fluids. However, replacement of the set involves manipulation of the circuit with potential for errors in asepsis and risk of contamination. A Cochrane review examined optimal replacement intervals ranging from 24 to 96 hours and found no difference in infection rates between the various replacement intervals [105]. International recommendations generally agree that administration sets do not have to be replaced routinely more frequently than every 96 hours [41, 43, 44].

RSVP is a recent non inferiority trial that compared a 4- versus 7-day interval for routine replacement of the administration sets. The 7-day interval was safe but only one AC-CRBSI was reported in the study [36]. Additionally, clinical judgement should be used and sets replaced if they become contaminated, faulty or blood cannot be cleared from the tubing.

*A scheduled change of AC administration sets every 7 days is safe unless clinically indicated earlier*.

#### Scheduled catheter changes

Scheduled catheter change may be an effective infection-prevention strategy for catheters that exhibit an increasing instantaneous risk of infection over time [106, 107]. In catheters placed with limited barrier precautions such as PIVCs (i.e. using a non-touch technique) it has been shown that instantaneous risk of CRBSI increases after 2 days of dwell time [106]. Although this has not been directly studied in ACs placed without MSBP, it is plausible that ACs which are placed using limited aseptic precautions, like PIVCs, would also exhibit an early rise in instantaneous risk. When considering ACs placed with MSBP, an older study showed that the daily risk of colonisation and AC-CRBSI increased significantly after 7 days while it remained constant for CVCs after the 5th day [3]. More frequently accessing ACs compared to CVCs may increase the risk of endoluminal colonisation over time [3]. More recent data in ACs placed with MSBP demonstrate an increase in AC-CRBSI only later (> 15 days) [37]. This might reflect improved care bundles implemented over the last decade.

Although not routinely recommended, scheduled change of ACs continues to be common practice in many ICUs worldwide [31]. The effectiveness of scheduled change as a preventive measure possibly depends on whether the insertion and postinsertion bundle for ACs are adhered to and needs further research.

*There is no evidence to support scheduled change of ACs for infection prevention within the current preventive care bundles*.

### Effective preventive measures and future research directions

**Fig. 2:**
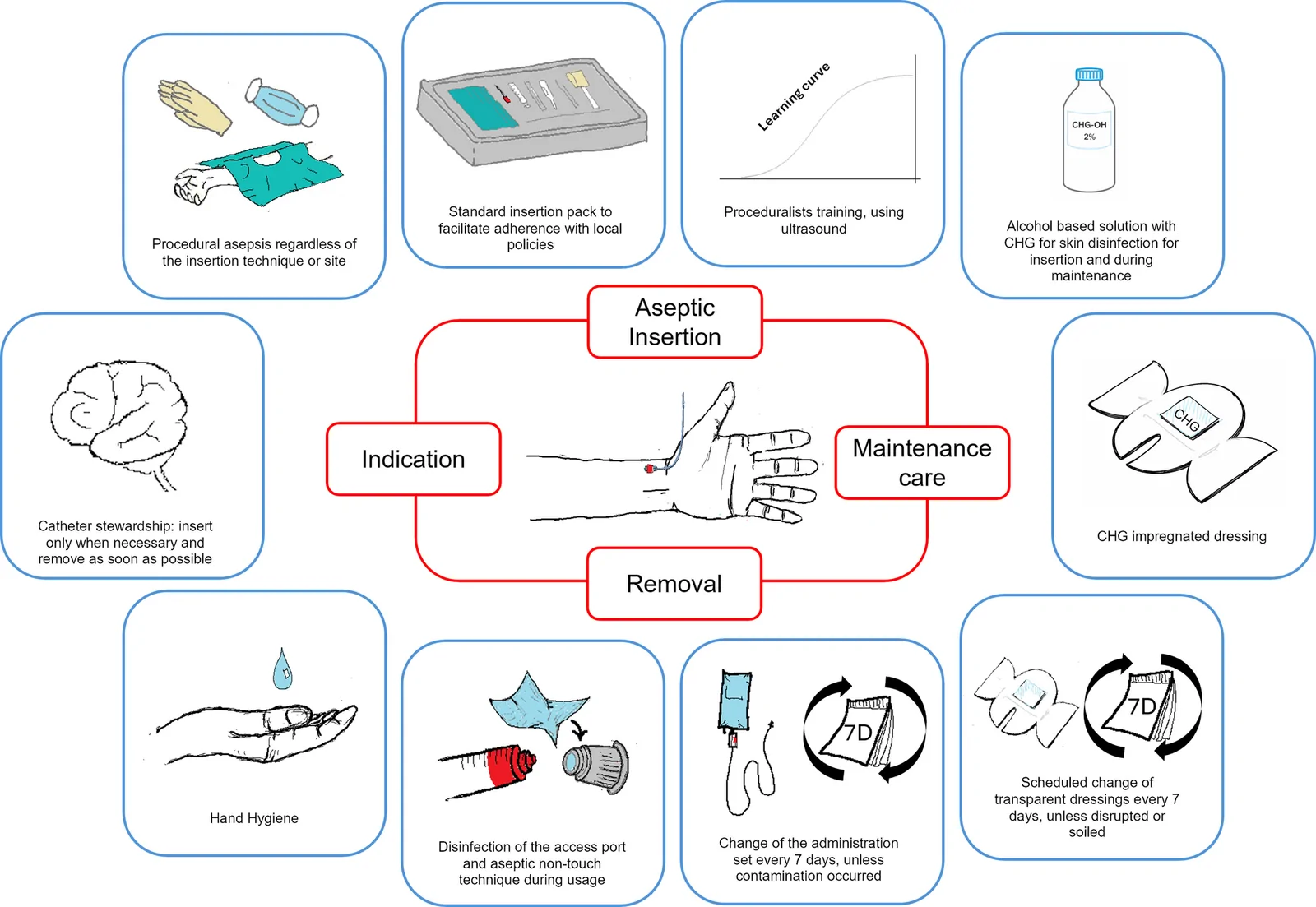
Ten suggested measures for the prevention of infection of arterial catheters in the ICU

Table 3 summarises current evidence on effective preventive measures and explores future research directions, highlighting ongoing gaps. Dedicated AC registries or adaptive platforms, similar to those already deployed for CVCs [108], could advance research more efficiently in the complex field of AC-CRBSI prevention. Figure 2 visually summarises ten effective preventive measures to reduce the incidence of AC-CRBSI in the ICU.

**Table 3:** Current evidence on infection prevention during the entire lifespan of arterial catheters, and future directions

	Current evidence	Future directions
**General measures**	Hand hygiene is effective for the prevention of CRBSI as part of a preventive bundle	Global educational campaigns to raise awareness among intensivists, anaesthetists, nurses and infectious disease specialists that ACs are an important source of BSIs Worldwide study/survey evaluating AC practices for insertion and maintenance Universal definitions to ensure comparable data to enforce surveillance and research on AC-CRBSI prevention
**Device stewardship**	ACs should be inserted only when clinically indicated and removed as soon as no longer necessary	Identification of patient groups benefitting most from AC insertion. Local quality improvement projects targeting stewardship. Testing of decision-making tools to prompt AC removal
**Site selection**	No given insertion site has been shown superior to another for infection prevention of AC-CRBSI	Large cohort study or randomised controlled trial comparing infectious risk between sites (femoral versus radial AC insertion). Multi-site trial to compare infectious risk between distal and proximal radial site insertions Site preference for specific subpopulations (e.g. obese patients)
**Barrier precautions**	Procedural asepsis as part of a care bundle during insertion is effective in limiting extraluminal colonisation and thus may be effective for the prevention of AC-CRBSI Optimal barrier precautions across various insertion techniques and settings of ACs remain uncertain	Delphi study to reach expert consensus on sterile barrier precautions with regards to device type/insertion technique and insertion setting Large cluster randomised trial, non-inferiority trial comparing SBP versus MSBP Consider cost-effectiveness and carbon footprint analysis
**US guidance**	Ultrasound guidance should be used for arterial cannulation independent of site selection to reduce mechanical complications and procedural time Its effect on infectious risk remains unknown	Prospective cohort to assess infectious risk with ultrasound use, correlate with clinicians’ proficiency/experience with ultrasound-guided arterial catheterisation Comparison of infection prevention practices during US guidance
**Training**	Clinicians’ training focusing on sterile procedural technique with ultrasound and continuous quality improvement programs are valuable strategies for infection prevention	Randomised study or quality improvement studies on simulation training for insertion, on post-insertion site care and competency assessment
**Cutaneous antisepsis**	Cutaneous antisepsis with a 2% CHG-alcohol solution is effective for infection prevention during insertion and maintenance of ACs	Effective alternatives for patients with CHG sensitivity. Surveillance for emergence of CHG resistance
**Insertion pack**	The use of standardised packs for AC insertion is a valuable strategy to facilitate adherence to guidelines and local protocols while improving procedural efficiency	Randomised multicentre study of AC insertion with a standard procedure pack and without one, including cost-effectiveness considerations
**Securement**	No particular securement device has been shown superior to another for the prevention of AC-CRBSI	Randomised multicentre study comparing different securement devices with infection endpoints, as well as mechanical complication (dislodgment, bleeding) endpoints
**Dressing type**	CHG-impregnated dressings are effective as part of a bundle to prevent AC-CRBSI	Quality improvement studies on the impact and cost-effectiveness of CHG-impregnated dressings depending on healthcare setting
**Dressing changes**	Unless soiled or disrupted, scheduled change of AC transparent dressings every 7 days is safe. Gauze dressings are changed every 1–2 days	Use of liquid medical adhesive to enhance dressing adherence
**Usage and access**	Thorough hand hygiene and disinfection of the access port before usage are key to reducing AC-CRBSI	Additional studies on optimal interventions to improve adhesion to hygiene practices and their effect on AC-CRBSI incidence. Comparative trials of closed and open systems including connectors to prevent infection
**Administration sets**	AC administration sets need not be changed more frequently than every 7 days unless clinically indicated	Non-inferiority trials with extended duration of administration sets, focusing on ACs
**Scheduled catheter changes**	There is no evidence to support scheduled change of ACs for infection prevention within the current preventive care bundle	Randomised study on scheduled catheter changes with regards to AC-CRBSI incidence

*AC* arterial catheter, *CVC* central venous catheter, *CLABSI* central line associated blood stream infection, *AC-CRBSI* arterial catheter related bloodstream infection, *CHG* chlorhexidine gluconate, *SBP* sterile barrier precautions, *MSBP* maximal sterile barrier precautions

## Conclusion

High quality evidence on the optimal measures for prevention of AC-CRBSI remain scarce and much remains extrapolated from CLABSI research. This likely contributes to the variability and, in some cases, the absence of specific recommendations for ACs across existing catheter-related infection prevention guidelines. Despite their widespread use, ACs carry an infectious risk that is often underrecognized by clinicians, therefore updated guidelines on the prevention of catheter infections that explicitly include ACs are needed. Innovative study designs, such as adaptive platform trials or dedicated AC registries, could help advance knowledge on optimal prevention strategies. While awaiting further evidence, available data should be drawn upon to reduce AC-related adverse events and improve the quality of care for critically ill patients.

There are numerous opportunities to reduce the infectious risk of ACs and its associated morbidity and mortality throughout the lifecycle of the catheter. In this review, in addition to highlighting gaps that need further research, we summarise existing literature related to AC infection prevention and suggest a set of effective preventive measures. In summary: proper stewardship regarding insertion and removal of ACs is crucial to limiting catheter days. Strict insertion bundles are effective in limiting extraluminal colonisation and a standardised insertion pack promotes adherence to an insertion bundle. Catheter insertion under ultrasound guidance limits mechanical complications related to arterial catheterisation, but the effect of ultrasound on catheter infection remains understudied. There is also no strong data to prefer an insertion site over another (i.e. radial over femoral). Hand hygiene, disinfection of the access port and aseptic non-touch technique during maintenance are fundamental elements of the AC care bundle. Cutaneous antisepsis with 2% CHG-alcohol solution is effective for infection prevention during insertion and maintenance of ACs. Adequate dressing care with prompt replacement of soiled dressings is important. Unless visibly disrupted or soiled, AC transparent dressings can safely be changed every 7 days. CHG-impregnated dressings have been shown effective as part of a bundle and superior to other options studied. Administration sets can safely be changed every 7 days. Finally, there is no evidence to support scheduled AC change within the current preventive bundle.

## Data Availability

No datasets were generated or analysed during the current study.

## Abbreviations

AC: Arterial catheter
AC-CRBSI: Arterial catheter-related bloodstream infection
ANTT: Aseptic non-touch technique
BSI: Bloodstream infection
CABSI: Catheter-associated bloodstream infection
CHG: Chlorhexidine gluconate
CLABSI: Central line-associated bloodstream infection
CRBSI: Catheter-related bloodstream infection
CVC: Central venous catheter
dTTP: Differential time to positivity
HOB: Hospital onset bacteraemia
ICU: Intensive care unit
MSBP: Maximal sterile barrier precautions
PIVC: Peripheral intravenous catheter
PiCC: Peripherally inserted central catheter
PVI: Povidone-iodine
SBP: Sterile barrier precautions
SSD: Sutureless securement device
US: Ultrasound
